# FCHO1 fine-tunes synaptic vesicle endocytosis in an activity-dependent manner

**DOI:** 10.1186/s13041-026-01295-1

**Published:** 2026-04-06

**Authors:** Hyun Jung Lee, Wongyoung Lee, Sung Hyun Kim

**Affiliations:** 1 Department of Neuroscience, Graduate School, Kyung Hee University, Seoul, 02447 South Korea; 2 Department of Physiology, School of Medicine, Kyung Hee University, Seoul, 02447 South Korea; 3 Department of Viral Disease, Animal and Plant Quarantine Agency, Gimcheon, 39660 South Korea; 4 School of Medicine, Medical Research Center for Bioreaction to Reactive Oxygen Species and Biomedical Science Institute, Kyung Hee University, Seoul, 02447 South Korea

**Keywords:** FCHO1, Synaptic vesicle endocytosis, Activity-dependent endocytosis, F-BAR, μ-HD

## Abstract

Synaptic vesicle (SV) recycling is critical for sustaining neurotransmission. Although FCHO1, a protein containing both an F-BAR domain and a μ-homology (μ-HD) domain, is recognized as a nucleator of clathrin-mediated endocytosis in non-neuronal systems, its physiological role at synapses remains unclear. Here, we investigated the function of FCHO1 in SV endocytosis at central synapses using a combination of shRNA-mediated knockdown and pHluorin-based live imaging. Within defined stimulation paradigms (25–300 action potentials at 10 Hz), depletion of FCHO1 markedly slowed endocytic kinetics across all stimulation intensities and was fully rescued by re-expression of an shRNA-resistant construct. Domain-specific functional analyses revealed stimulation-strength-dependent functional requirements. The F-BAR domain was sufficient to support vesicle retrieval under low stimulation conditions, whereas the μ-homology domain (μ-HD) became essential as stimulation strength increased. These findings support a model in which FCHO1 operates as a demand-sensitive scaffold within the endocytic pathway, with distinct structural domains differentially required as neural activity and consequently endocytic load escalates. Our results establish FCHO1 as a critical regulator of SV endocytosis and suggest that multidomain endocytic proteins may scale their functional contributions according to the magnitude of neuronal activation.

## Introduction

Neurons communicate through synaptic transmission, a tightly regulated process in which neurotransmitters are released from presynaptic terminals and detected by postsynaptic receptors. This process relies on a dynamic balance between synaptic vesicle (SV) exocytosis and endocytosis, ensuring the sustained availability of vesicles for continued neurotransmission [1, 2]. Upon arrival of an action potential (AP), calcium influx into the presynaptic boutons triggers SV fusion with the plasma membrane, resulting in neurotransmitter release [3, 4]. To maintain synaptic function, SV must be rapidly retrieved via endocytosis for reuse.

Multiple modes of SV endocytosis have been described, including clathrin-mediated endocytosis (CME), ultrafast endocytosis, bulk endocytosis, and kiss-and-run [5–8] Among these, CME is considered one of the most well-known mechanisms at most central synapses. During CME, BAR domain-containing proteins recognize PI(4,5)P_2_ at the plasma membrane and initiate membrane curvature to form clathrin-coated pits [9]. Clathrin assembly and dynamin-mediated membrane fission complete vesicle retrieval, followed by neurotransmitter reloading into regenerated vesicles [10, 11]. Disruption of SV endocytosis is associated with synaptic dysfunction and has been implicated in neurodegenerative diseases such as Huntington’s and Parkinson’s diseases [12].

Among the molecular players in CME, FCHo1 and FCHo2 (Fes/CIP4 homology domain-only proteins) were identified as pioneering nucleators of endocytosis [13, 14]. These F-BAR domain-containing proteins consist of three major regions: an N-terminal F-BAR domain, a central intrinsically disordered linker region, and a C-terminal μ-homology domain (μ-HD) [15, 16]. FCHO1 is enriched in the brain and spleen, while FCHO2 is more broadly expressed [17, 18]. The F-BAR domain induces shallow membrane curvature through PI(4,5)P_2_ recognition [19], the linker domain stabilizes AP-2 via direct interactions [20, 21], and the μ-HD domain mediates recruitment of endocytic adaptors such as Eps15 and intersectin by recognizing DPF motifs [15, 22].

FCHO1 is thus positioned at the top of the endocytic hierarchy, acting as a scaffold for clathrin coat assembly by both recruiting and activating AP-2 [21–24]. However, while its mechanistic roles in non-neuronal cells are relatively well established, its physiological significance at synapses remains poorly characterized.

Importantly, synaptic vesicle recycling is not static—it is dynamically regulated by the intensity and frequency of neuronal activity [25] For example, vesicle retrieval is notably slower both at low stimulation (e.g., 25 APs) and during prolonged high-frequency firing (e.g., 300 APs), possibly reflecting differential demands on the endocytic machinery [25–27]. These observations raise the possibility that specific components of the endocytic pathway may play activity-dependent roles.

In this study, we investigated how FCHO1 contributes to synaptic vesicle endocytosis across different levels of neuronal activity. We reveal that distinct domains of FCHO1—the F-BAR and μ-HD regions—differentially regulate endocytosis under low and high activity, respectively. These findings support a model in which FCHO1 fine-tunes synaptic vesicle endocytosis in an activity-dependent and domain-specific manner.

## Materials and methods

### Primary neuron culture

Hippocampal CA3–CA1 regions were isolated from 0–1-day-old Sprague Dawley rats (DBL, strain code: NTac:SD), dissociated, and plated onto poly-ornithine-coated coverslips. Neurons were transfected on day 8 in vitro and maintained in culture for a total of 14–21 days. Live-cell imaging was conducted between days 14 and 21 post-plating. All experimental data were obtained from at least three independent primary cultures. All animal procedures were conducted in accordance with institutional guidelines and approved by the Animal Care Committee of Kyung Hee University.

### Plasmids and transfection

A short hairpin RNA (shRNA) targeting FCHO1 (referred to as FCHO1-KD), corresponding to the sequence 5′-GCAGGTGCATGAGGAATTTAA-3′, was synthesized, annealed, and cloned into the pSuper vector, as previously described [25]. Domain-deletion mutants of FCHO1 were generated by PCR using specific primers and an shRNA-resistant FCHO1 template. These mutant constructs were cloned in-frame into a modified pEGFP-N1 vector in which the EGFP coding region was replaced with mCherry. For co-expression experiments, the constructs were also subcloned into a PT2A bicistronic plasmid allowing co-expression of vGlut-pHluorin (vG-pH) and the FCHO1 variants. Primary hippocampal neurons were transfected using the calcium phosphate precipitation method, following the protocol described by Kim and Ryan [28]. Plasmid DNA was mixed with 2 mM CaCl_2_ and 2 × HeBS buffer (273 mM NaCl, 9.5 mM KCl, 1.4 mM Na_2_HPO_4_·7H_2_O, 15 mM D-glucose, 42 mM HEPES, pH 7.10), and the resulting DNA-calcium phosphate precipitate was applied to neuronal cultures.

### Immunofluorescence

For immunofluorescence analysis, neurons at DIV14–21 were fixed with 4% paraformaldehyde (PFA), permeabilized using 0.2% Triton X-100, and blocked with 5% bovine serum albumin (BSA). Neurons were then incubated overnight at 4 °C with primary anti-FCHO1 antibodies (Thermo). Following primary antibody incubation, cells were treated with Alexa Fluor 488- or Alexa Fluor 546-conjugated secondary antibodies (Invitrogen), selected according to the desired fluorescence channel combination.

### Optical imaging

Images of fixed cells were acquired using a Leica DMRBE microscope equipped with a PL Fluotar 40 × objective lens (1.0 NA; Leica) and a CoolSNAP HQ camera (Photometrics), controlled by MetaMorph software. For live imaging of presynaptic terminals to assess synaptic vesicle recycling, neurons were transfected at 8 days in vitro (DIV) with vG-pH constructs, with or without shRNA-FCHO1-KD1 (see ‘Plasmids’ in Methods). Experiments were conducted at DIV14–21 using mature neurons. Coverslips were mounted in a laminar-flow perfusion stimulation chamber placed on the stage of a custom-built laser-illuminated epifluorescence microscope (Zeiss Observer). Live-cell fluorescence images were captured with an Andor iXon Ultra 897 back-illuminated EMCCD camera (Model #DU-897U-CS0-#BV). A Cobolt 488 or 561 diode-pumped solid-state laser was used for excitation, synchronized with the EMCCD’s TTL on/off signal during acquisition. Fluorescence excitation and detection were performed using a 40 × Fluar objective lens (1.3 NA; Zeiss), with a 498 nm dichroic mirror and 500–550 nm emission filter (Chroma) for pHluorin imaging. Action potentials (APs) were evoked by delivering 1-ms current pulses through platinum-iridium electrodes using an isolated current stimulator (World Precision Instruments). Neurons were perfused with Tyrode’s solution containing 119 mM NaCl, 2.5 mM KCl, 2 mM CaCl_2_, 2 mM MgCl_2_, 25 mM HEPES, 30 mM glucose, 10 μM CNQX, and 50 μM D,L-AP5 (pH adjusted to 7.4). All recordings were performed at 30 °C. To alkalinize acidic compartments, 50 mM NH_4_Cl was substituted for 50 mM NaCl (pH 7.4). Stimulation protocols for vG-pH imaging included 2.5, 5, 10, and 30 s stimulation trains at 10 Hz, with or without co-transfected plasmids. NH_4_Cl was applied at the end of each recording to reveal the total recycling pool. Images were acquired at 2 Hz with 50-ms exposure time. To assess synaptic vesicle exocytosis rates and recycling pool size, bafilomycin A1 (100 μM; Calbiochem) was applied 30 s prior to image acquisition.

### Imaging analysis

Image analysis was performed using ImageJ (https://imagej.nih.gov/ij) with the Time Series Analyzer plugin (https://imagej.nih.gov/ij/plugins/time-series.html). FCHO1 distribution at synaptic boutons was quantified by identifying vGlut1-pHluorin–positive boutons (vG-pH) in ImageJ. pHluorin signal analysis was conducted following previously described protocols [29], with minor modifications. To assess differences in FCHO1 expression with or without shRNA-FCHO1, fluorescence intensity was measured in synaptic boutons selected as oval regions of interest (ROIs; 10-pixel diameter). Fluorescence intensity values were quantified in ImageJ and further analyzed using OriginPro (version 2020). Endocytic kinetics were fitted to a single-exponential decay model.

### Statistical analysis

Statistical analyses were conducted using either one-way analysis of variance (ANOVA) or Student’s *t*-test, as appropriate. Data are presented as mean ± standard error of the mean (SEM), and error bars represent SEM.

## Results

### FCHO1 is localized at presynaptic nerve terminals in hippocampal neurons.

**Fig. 1:**
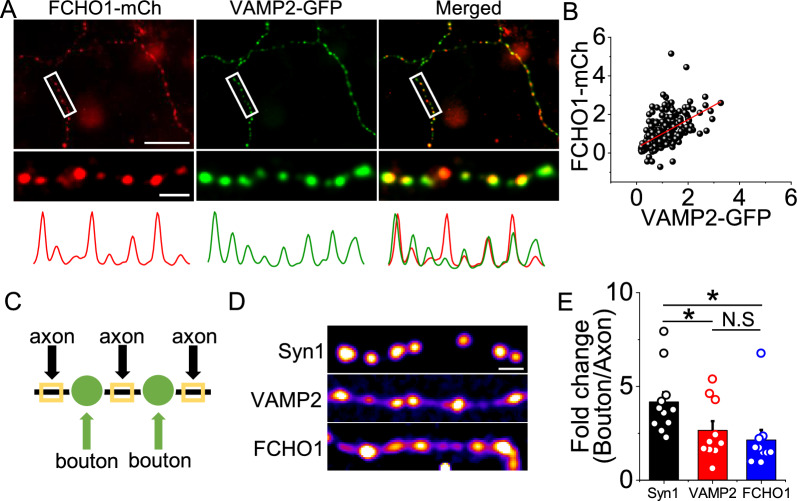
FCHO1 localizes at nerve terminals of primary cultured neurons. **a** Representative images of cultured hippocampal neurons co-expressing FCHO1-mCh (left) and the presynaptic marker VAMP2-GFP (right). Neurons were transfected at DIV8 and fixed at DIV14-16. Scale bars: 10 μm (top), 5 μm (bottom). **b** Quantification of the fluorescence intensity correlation between FCHO1-mCherry and VAMP2-GFP. **c** Representative image highlighting axons (black) and presynaptic boutons (green). A schematic indicates regions of interest (ROI) selected for bouton-versus-axon analysis. **d** Representative images showing the distribution of Synapsin1-GFP, VAMP2-GFP, and FCHO1-mCherry along axons and dendrites. Scale bar: 5 μm. **e** Quantification of fluorescence enrichment at boutons relative to axons. Fold changes (bouton/axon) are as follows: Synapsin1-GFP = 4.17 ± 0.52 (n = 13 cells, VAMP2-GFP = 2.66 ± 0.50 (n = 10 cells), FCHO1-mCherry = 2.15 ± 0.54 (n = 10 cells). *p < 0.05

FCHO1, a member of the F-BAR domain-containing protein family, is known to act as a nucleator of clathrin-mediated endocytosis (CME) [15]. However, its specific role at synapses remains poorly understood. To investigate whether FCHO1 is involved in synaptic vesicle endocytosis, we examined its subcellular localization in relation to the presynaptic marker VAMP2. Hippocampal neurons were co-transfected with FCHO1-GFP and VAMP2-mCherry, and their distribution was visualized using fluorescence microscopy. We found that FCHO1 co-localized with VAMP2 (Fig. 1a), and their fluorescence intensities showed a positive correlation (Fig. 1b), suggesting that FCHO1 is present at presynaptic terminals. To further assess the specificity of FCHO1 localization at synaptic boutons, we compared its distribution with the synaptic proteins synapsin1 and VAMP2 along axons and at synapses. FCHO1 displayed a synaptic distribution pattern similar to that of VAMP2 (Fig. 1c–e), supporting the notion that FCHO1 is localized at presynaptic nerve terminals.

### Depletion of FCHO1 alters the rate of synaptic vesicle endocytosis after neural activities.

**Fig. 2:**
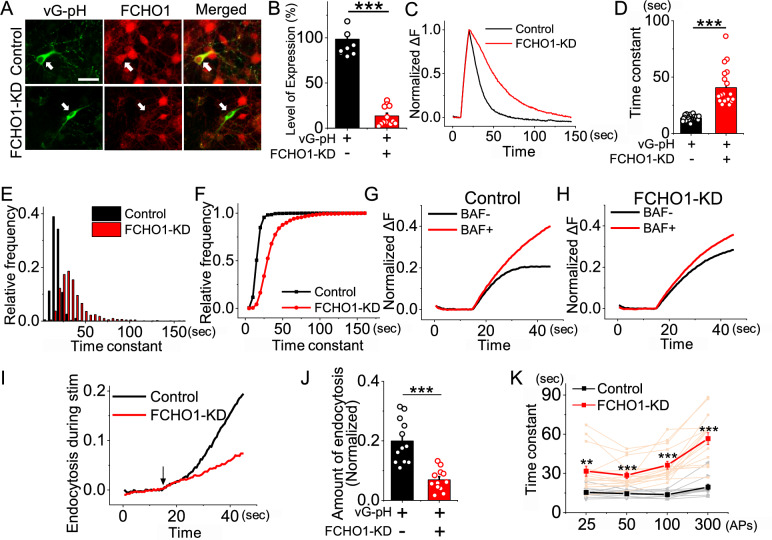
FCHO1 depletion impairs synaptic vesicle endocytosis in response to neuronal stimulation. **a** Representative images of primary hippocampal neurons transfected with vGlut1-pHluorin (vG-pH) with or without shRNA targeting FCHO1 at DIV8 and immunostained for endogenous FCHO1 (red) at DIV15. Scale bar = 5 μm. **b** Mean value of FCHO1 expression in control and FCHO1-KD neurons. Expression was significantly reduced in FCHO1-KD cells [FCHO1]_con_ = 98.53 ± 4.98 (n = 8 cells), [FCHO1]_FCHO1-KD_ = 13.65 ± 3.00 (n = 13 cells). **c** Representative trace of the vG-pH response at 100 APs from Control (Black) and FCHO1-KD (Red) neurons. **d** Mean value of post-stimulus endocytosis time constants from Control (τ_endo_ = 13.65 ± 0.52 s; n = 25 cells) and FCHO1-KD (τ_endo=_ 40.76 ± 3.49 s; n = 20 cells) neurons. **e–f** Distribution of endocytic time constants across individual synaptic boutons in control (black) and FCHO1-KD (red) neurons: **e** relative frequency histogram, **f** cumulative distribution. **g–h** Representative traces of the vG-pH response to 300 APs at 10 Hz with (red)/without (black) BAF (Bafilomycin A1) from Control (**g**) and (**h**) FCHO1-KD neurons. **i** Representative normalized traces showing the amount of endocytosis during stimulation in control (black) and FCHO1-KD (red) neurons. **j** Endocytosis during stimulus, calculated as ΔF_Baf_ − ΔF_300 non Baf_ and normalized to the NH_4_Cl responses. [FCHO1]_Con_ = 0.20 ± 0.02 (n = 12 cells), [FCHO1]_FCHO1-KD_ = 0.07 ± 0.01 (n = 12 cells). **k** Endocytosis time constants measured after varying stimulation intensities (25, 50, 100, and 300 APs): Control = 15.61 ± 1.11 s (25 APs), 14.67 ± 0.93 s (50 APs), 13.88 ± 0.77 s (100 APs), 19.67 ± 2.20 s (300 APs), n = 11 cells; FCHO1-KD = 31.88 ± 3.85 s (25 APs), 28.69 ± 2.45 s (50 APs), 36.47 ± 3.00 s (100 APs), 56.78 ± 4.58 s (300 APs), n = 16 cells. **p < 0.01, ***p < 0.001

Having confirmed the presence of FCHO1 at presynaptic nerve terminals—and given its previously established role as a nucleator of clathrin-mediated endocytosis (CME) in non-neuronal cells [15]—we next investigated whether FCHO1 plays a functional role in synaptic vesicle endocytosis. To this end, we constructed an shRNA targeting FCHO1 and transfected it into primary cultured hippocampal neurons. Due to the inherently low transfection efficiency in primary neurons, conventional biochemical approaches such as western blotting are suboptimal for assessing knockdown efficiency. Instead, we employed immunocytochemistry to quantify FCHO1 expression at the single-cell level. This analysis revealed a substantial reduction in FCHO1 expression, down to 13.63% of control levels (Fig. 2a, b).

To monitor synaptic vesicle recycling, we utilized a well-established pHluorin-based assay [25, 30, 31]. Neurons were co-transfected with vGlut1-pHluorin (vG-pH) and shRNA against FCHO1 (FCHO1-KD), and then stimulated with 100 action potentials (APs). The rate of synaptic vesicle endocytosis was markedly reduced in FCHO1-KD neurons, with the time constant of endocytosis increasing more than three-fold compared to control (WT = 13.65 ± 0.52 s, FCHO1-KD = 40.76 ± 3.49 s) (Fig. 2c, d). At the level of individual synaptic boutons, endocytosis kinetics exhibited a similar pattern of deceleration (Fig. 2e, f).

To determine whether this impairment persisted during ongoing neural activity, we applied prolonged stimulation (300 APs at 10 Hz) in the presence or absence of bafilomycin A1 (BAF), a specific inhibitor of the vacuolar-type H^+^-ATPase [32]. By comparing vG-pHluorin fluorescence traces with and without BAF, we were able to isolate and quantify endocytosis occurring during stimulation [33]. This analysis revealed that FCHO1 knockdown significantly reduced the amount of endocytosis during sustained activity compared to controls (Fig. 2g–j).

We further extended this approach by examining a range of stimulation protocols (25, 50, 100, and 300 APs), as synaptic vesicle endocytosis varies with stimulation strength [25, 34]. Under all stimulation conditions, FCHO1 knockdown consistently resulted in significantly slower endocytosis kinetics (Fig. 2k). These results indicate that FCHO1 is broadly required for efficient synaptic vesicle endocytosis across stimulation intensities.

### Reintroduction of FCHO1 restores synaptic vesicle endocytosis in FCHO1-KD neurons

To determine whether the observed endocytic defects were specifically due to the loss of FCHO1 rather than off-target effects of the shRNA, we employed a shRNA-resistant construct encoding FCHO1 fused to mCherry (FCHO1-mCh res), as described previously [35]. Hippocampal neurons were co-transfected with vG-pH, FCHO1-targeting shRNA, and FCHO1-mCh^res, and were subjected to synaptic stimulation to assess vesicle endocytosis.

**Fig. 3:**
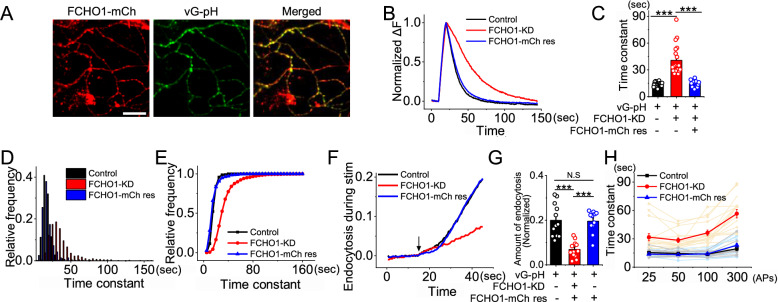
Rescue of impaired synaptic vesicle endocytosis by re-expression of shRNA-resistant FCHO1. **a** Representative images of neuron transfected with vG-pH, shRNA-resistant FCHO1 at DIV8 and imaged 7-8 days later. scale bar = 5 μm. **b** Representative trace of the vG-pH in response to 100 APs from Control (black), FCHO1-KD (red) and FCHO1-rescue (blue) neurons. **c** Mean value of post-stimulus endocytosis time constants from Control (τ_endo_ = 13.66 ± 0.52 s; n = 20 cells), FCHO1-KD (τ_endo=_40.76 ± 3.69 s; n = 20 cells) and FCHO1-rescue (τ_endo_ = 14.25 ± 0.97 s; n = 15 cells) neurons. **d–e** Distribution of endocytic time constants from single synaptic bouton analysis in each group: **d** relative frequency, **e** cumulative distribution. **f** Representative traces of control (black) and FCHO1-KD (red) neurons **g** Mean value of the amount of endocytosis during stimulation in Control (black), FCHO1-KD (red) and FCHO1-rescue (blue) neurons. Endocytosis during stimulus, calculated as ΔF_Baf_ − ΔF_300 non Baf_ and normalized to the NH_4_Cl responses. [FCHO1]_Con_ = 0.20 ± 0.02 (n = 12 cells), [FCHO1]_FCHO1-KD_ = 0.07 ± 0.01 (n = 12 cells), [FCHO1]_FCHO1-Rescue_ = 0.20 ± 0.02 (n = 11 cells). **h** Synaptic vesicle endocytosis time constants measured after different levels of stimulation (25–300 APs): Control (25 APs = 15.61 ± 1.11 s, 50 APs = 14.67 ± 0.93 s, 100 APs = 13.88 ± 0.77 s, 300 APs = 19.67 ± 2.2 s; n = 11 cells), FCHO1-KD (25 APs = 31.88 ± 3.85 s, 50 APs = 28.69 ± 2.45 s, 100 APs = 36.47 ± 3.00 s, 300 APs = 56.78 ± 4.58 s; n = 16 cells) and FCHO1-rescue (25 APs = 13.97 ± 0.87 s, 50 APs = 13.46 ± 0.80 s, 100 APs = 14.25 ± 0.97 s, 300 APs = 23.35 ± 2.11 s; n = 15 cells). ***p < 0.001

Neurons expressing the shRNA-resistant FCHO1-mCh res construct (Fig. 3a) exhibited a substantial restoration of synaptic vesicle endocytosis kinetics, returning to levels comparable to those of control neurons (Fig. 3b–e). Additionally, other endocytic processes, including those occurring during stimulation and under varying stimulation intensities, were also robustly rescued to control levels (Fig. 3f–h). These results strongly support the conclusion that FCHO1 is directly involved in regulating synaptic vesicle endocytosis.

### The F-BAR Domain of FCHO1 is essential for synaptic vesicle endocytosis under low neural activity

FCHO1 consists of three structurally distinct regions: two conserved domains—the F-BAR (Fes-CIP4 Homology-Bin/Amphiphysin/Rvs) domain and the μ-homology domain (μ-HD)—and a linker region that connects them. The F-BAR domain is known to generate shallow, positively curved membrane structures and functions as a scaffold for the assembly of endocytic machinery [13, 14, 17, 36]. Although the general function of the F-BAR domain is well established, its specific physiological relevance in the context of FCHO1-mediated synaptic vesicle endocytosis remains unclear.

**Fig. 4:**
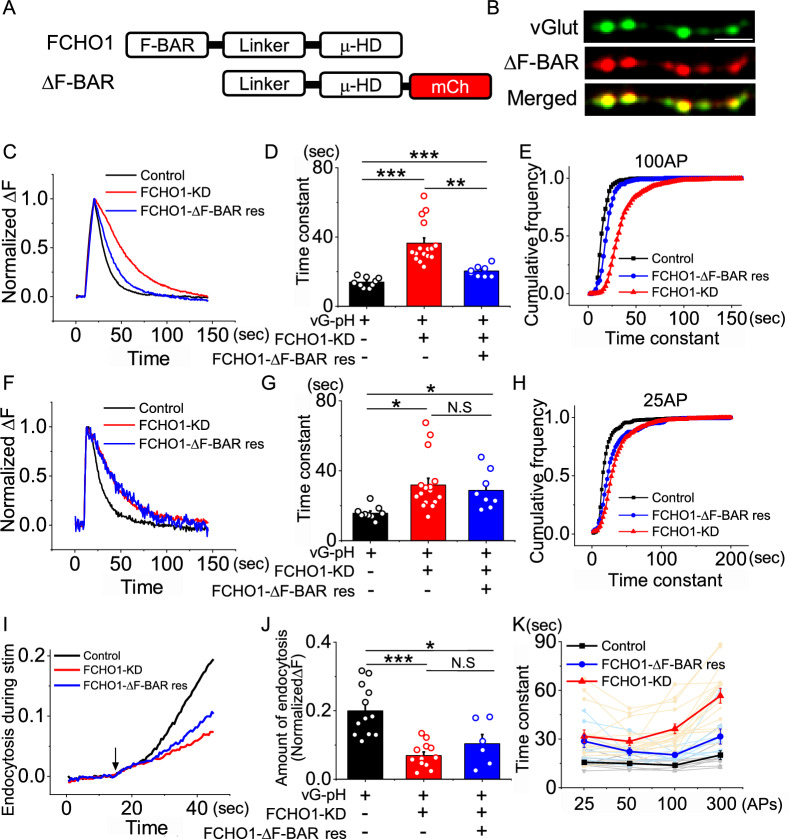
The F-BAR domain of FCHO1 is required for efficient synaptic vesicle endocytosis under low stimulation conditions. **a** Schematic diagram of F-BAR domain deletion mutant of FCHO1. **b** Deletion mutants of FCHO1 F-BAR domain localizes at presynaptic terminals in cultured hippocampal neuron. Scale bar = 5 μm. **c** Representative trace of the vG-pH in response to 100AP from Control (black), FCHO1-KD (red) and ΔF-BAR (blue) neurons. **d** Mean value of post-stimulus endocytosis time constants at 100 APs from Control (τ_endo_ = 13.98 ± 0.94 s; n = 9 cells), FCHO1-KD (τ_endo=_36.47 ± 3.00; n = 16 cells) and ΔF-BAR (τ_endo_ = 20.38 ± 1.13 s; n = 8 cells) neurons. **e** Cumulative frequency of time constants from individual bouton analysis under 100 APs stimulation in control (black), FCHO1-KD (red) and ΔF-BAR (blue) neurons **f** Representative trace of the vG-pH in response to 25 APs from control (black), FCHO1-KD (red) and ΔF-BAR rescue (blue) neurons. **g** Mean value of post-stimulus endocytosis time constants at 25 APs from Control (τ_endo_ = 15.76 ± 1.25 s; n = 9 cells), FCHO1-KD (τ_endo=_31.88 ± 3.85 s; n = 16 cells), and ΔF-BAR rescue (τ_endo_ = 28.79 ± 3.82 s; n = 8 cells) neurons. **h** Cumulative frequency of time constants from individual bouton analysis under 25 APs stimulation in control (black), FCHO1-KD (red) and ΔF-BAR rescue (blue) neurons. **i** Representative traces of Control (black), FCHO1-KD (red) and ΔF-BAR rescue (blue) neurons during sustained stimulation **j** Quantification of endocytosis during stimulation, calculated as ΔF_Baf-_ − ΔF_300 non Baf_ and normalized to the NH_4_Cl responses. [FCHO1]_Con_ = 0.20 ± 0.02 (n = 12 cells), [FCHO1]_FCHO1-KD_ = 0.07 ± 0.01 (n = 12 cells), [FCHO1]_ΔF-BAR rescue_ = 0.10 ± 0.03 (n = 6 cells). **k** Endocytosis time constants after various stimulation conditions: control (25 APs = 15.76 ± 1.25 s, 50 APs = 15.08 ± 1.06 s, 100 APs = 13.98 ± 0.94 s, 300 APs = 20.23 ± 2.68 s; n = 9 cells), FCHO1-KD (25 APs = 31.88 ± 3.85 s, 50 APs = 28.69 ± 2.45 s, 100 APs = 36.47 ± 3.00 s, 300 APs = 56.78 ± 4.58 s; n = 8 cells) and ΔF-BAR rescue (25 APs = 28.79 ± 3.83 s, 50 APs = 22.41 ± 2.32 s, 100 APs = 20.38 ± 1.3 s, 300 APs = 31.70 ± 4.63 s; n = 16 cells). *p < 0.05, **p < 0.01, ***p < 0.001

To address this, we generated a deletion mutant of FCHO1 lacking the F-BAR domain (ΔF-BAR, Fig. 4a) and confirmed its expression and synaptic localization (Fig. 4b). We then replaced endogenous FCHO1 with FCHO1-ΔF-BAR by co-transfecting FCHO1 shRNA and the deletion construct into hippocampal neurons. Synaptic vesicle endocytosis was assessed under various physiological stimulation conditions.

Under standard stimulation (100 APs at 10 Hz), neurons expressing FCHO1-ΔF-BAR exhibited a moderate but statistically significant slowing of endocytosis (20.38 ± 1.13 s, Fig. 4c–e). Interestingly, under low stimulation conditions (25 APs at 10 Hz), endocytosis was severely impaired (28.79 ± 3.82 s), comparable to that observed in FCHO1 knockdown neurons (31.88 ± 3.86 s, Fig. 4f–h, k). These findings indicate that the relative contributions of the F-BAR domain is more pronounced under low stimulation strength. Consistent with this, the amount of endocytosis occurring during prolonged stimulation was also markedly reduced in FCHO1-ΔF-BAR-expressing neurons (Fig. 4i, j), suggesting that the absence of the F-BAR domain alone is not dominant negative under basal conditions.

**Fig. 5:**
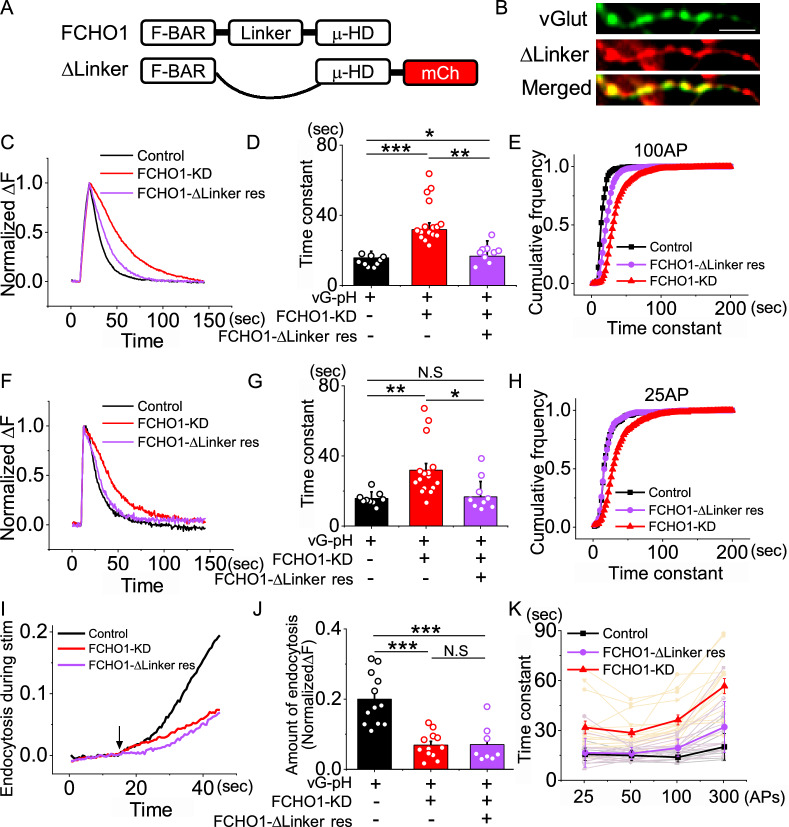
The linker domain of FCHO1 contributes modestly to synaptic vesicle endocytosis during neuronal stimulation. **a** Schematic diagram of the FCHO1 construct lacking the central linker domain (ΔLinker). **b** ΔLinker mutant FCHO1 localizes to presynaptic sites in cultured hippocampal neurons. Scale bar = 5 μm. **c** Representative trace of the vG-pH in response to 100 APs from control (black), FCHO1-KD (red) and ΔLinker (purple) neurons. **d** Mean value of post-stimulus endocytosis time constants at 100 APs from control (τ_endo_ = 13.98 ± 0.94 s; n = 9 cells), FCHO1-KD (τ_endo=_36.47 ± 3; n = 16 cells) and ΔLinker rescue (τ_endo_ = 18.72 ± 1.76 s; n = 9 cells) neurons. **e** Cumulative frequency of time constants from single synaptic bouton analysis under 100 APs stimulation: control (black), FCHO1-KD (red) and ΔLinker (purple) neurons. **f** Representative trace of the vG-pH in response to 25 APs from control (black), FCHO1-KD (red) and ΔLinker (purple) neurons. **g** Mean value of post-stimulus endocytosis time constants at 25 APs from control (τ_endo_ = 15.76 ± 1.25 s; n = 9 cells), FCHO1-KD (τ_endo_ = 31.88 ± 3.86 s; n = 16 cells) and ΔLinker rescue (τ_endo_ = 18.31 ± 3.2 s; n = 9 cells) neurons. **h** Cumulative frequency of time constants from single synaptic bouton analysis under 25 APs stimulation: control cell (black), FCHO1-KD (red) and ΔLinker rescue (purple) neurons. **i** Representative traces of endocytosis during sustained stimulation: control (black), FCHO1-KD (red) and ΔLinker rescue (purple) neurons **j** Quantification of endocytosis during stimulation, calculated as ΔF_Baf_-ΔF_300 non Baf_ and normalized to the NH_4_Cl responses. [FCHO1]_Con_ = 0.20 ± 0.02 (n = 12 cells), [FCHO1]_FCHO1-KD_ = 0.07 ± 0.01 (n = 12 cells), [FCHO1]_ΔLinker rescue_ = 0.07 ± 0.02 (n = 8 cells). **k** Endocytosis time constants across various stimulation conditions: control (25 APs = 15.76 ± 3.76 s, 50 APs = 15.08 ± 3.17 s, 100 APs = 13.98 ± 2.83 s, 300 APs = 20.23 ± 8.04 s; n = 9 cells), FCHO1-KD (25 APs = 31.88 ± 3.85 s, 50 APs = 28.69 ± 2.45 s, 100 APs = 36.47 ± 3.00 s, 300 APs = 56.78 ± 4.58 s; n = 16 cells) and ΔLinker rescue (25 APs = 16.72 ± 8.71 s, 50 APs = 16.23 ± 3.64 s, 100 APs = 19.59 ± 5.25 s, 300 APs = 32.17 ± 15.31 s; n = 13 cells). *p < 0.05, **p < 0.01, ***p < 0.001

We next examined the role of the linker region in FCHO1-mediated endocytosis using a similar approach. Endogenous FCHO1 was replaced with a construct lacking the linker region (FCHO1-ΔLinker), and synaptic vesicle trafficking was monitored. While endocytosis was slightly slowed (19.59 ± 5.26 s), no major defects were observed across various stimulation paradigms, including sustained stimulation (Fig. 5), indicating that the linker region is not essential for synaptic vesicle endocytosis.

### The μ-HD Domain of FCHO1 is required for synaptic vesicle endocytosis during prolonged neural activity

The μ-homology domain (μ-HD) of FCHO1 is structurally similar to the μ subunit of the AP-2 complex and is thought to serve as a protein–protein interaction hub. Specifically, μ-HD recruits essential components of the clathrin-mediated endocytic machinery—such as Eps15, Intersectin, and AP-2—by recognizing DPF motifs on these proteins [20, 37]. Based on these known interactions, we hypothesized that the μ-HD domain of FCHO1 is essential for synaptic vesicle endocytosis by acting as a critical scaffolding and recruitment region.

**Fig. 6:**
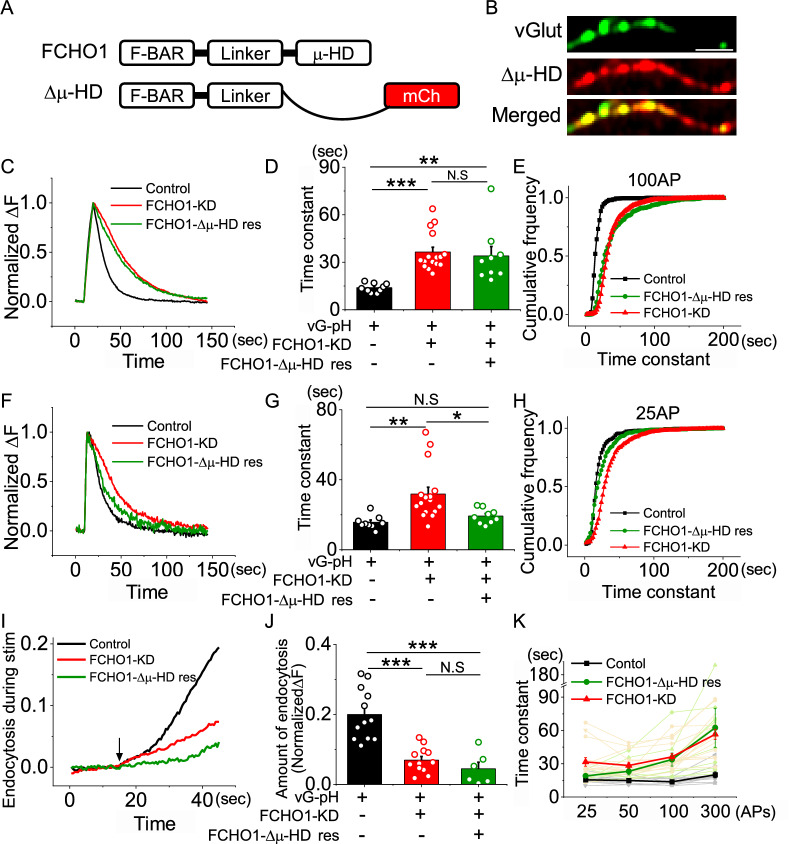
μ-HD domain of FCHO1 are influenced for synaptic vesicle retrieval at bulk stimulus. **a** Schematic diagram of the FCHO1 construct lacking the μ-HD domain (Δμ-HD). **b** The Δμ-HD mutant localizes properly to presynaptic terminals in primary cultured hippocampal neurons. Scale bar = 5 μm. **c** Representative trace of the vG-pH in response to 100 APs from control (black), FCHO1-KD (red) and Δμ-HD (green) neurons. **d** Mean value of post-stimulus endocytosis time constants at 100 APs from control (τ_endo_ = 13.98 ± 0.94 s; n = 9 cells), FCHO1-KD (τ_endo=_36.47 ± 3; n = 16 cells) and Δμ-HD rescue (τ_endo_ = 34.04 ± 5.89 s; n = 9 cells) neurons. **e** Cumulative frequency of time constants under 100 APs stimulation from single synaptic bouton analysis in control (black), FCHO1-KD (red) and Δμ-HD rescue (green) neurons. **f** Representative trace of the vG-pH in response to 25 APs from control (black), FCHO1-KD (red) and Δμ-HD rescue (green) neurons. **g** Mean value of post-stimulus endocytosis time constants at 25 APs from control (τ_endo_ = 15.76 ± 1.25 s; n = 9 cells), FCHO1-KD (τ_endo=_31.88 ± 3.85 s; n = 16 cells) and Δμ-HD rescue (τ_endo_ = 19.27 ± 1.40 s; n = 9 cells) neurons. **h** Cumulative frequency of time constants under 25 APs stimulation from single synaptic bouton analysis in control (black), FCHO1-KD (red) and Δμ-HD rescue (green) neurons. **i** Representative traces showing endocytosis during sustained stimulation: control (black), FCHO1-KD (red) and Δμ-HD rescue (green) neurons **j** Quantification of endocytosis during stimulation, measured as ΔF_Baf_-ΔF_300 non Baf_ and normalized to the NH_4_Cl responses. [FCHO1]_Con_ = 0.20 ± 0.02 (n = 12 cells), [FCHO1]_FCHO1-KD_ = 0.07 ± 0.01 (n = 12 cells), [FCHO1]_Δ_μ_-HD rescue_ = 0.04 ± 0.02 (n = 6 cells). **k** Endocytosis time constants across various stimulation intensities: control (25 APs = 15.75 ± 1.25 s, 50 APs = 15.08 ± 1.06 s, 100 APs = 13.98 ± 0.94 s, 300 APs = 20.23 ± 2.68 s; n = 9 cells), FCHO1-KD (25 APs = 31.88 ± 3.85 s, 50 APs = 28.69 ± 2.45 s, 100 APs = 36.47 ± 3.00 s, 300 APs = 56.78 ± 4.58 s; n = 16 cells) and Δμ-HD rescue (25 APs = 19.27 ± 1.40 s, 50 APs = 23.41 ± 3.16 s, 100 APs = 34.04 ± 5.89 s, 300 APs = 62.68 ± 17.61 s; n = 9 cells). *p < 0.05, **p < 0.01, ***p < 0.005

To test this, we generated a deletion construct lacking the μ-HD domain (FCHO1-Δμ-HD) and replaced endogenous FCHO1 with this mutant (Fig. 6a, b). Synaptic vesicle endocytosis was then monitored under various stimulation paradigms. Notably, under standard stimulation conditions (100 APs at 10 Hz), neurons expressing FCHO1-Δμ-HD failed to rescue the endocytosis defect, exhibiting kinetics nearly identical to those of FCHO1 knockdown neurons (Fig. 6c–e). Similarly, endocytosis during sustained stimulation was also severely impaired in FCHO1-Δμ-HD-expressing neurons (Fig. 6i, j).

In contrast, under low stimulation conditions (25 APs at 10 Hz), endocytosis kinetics appeared comparable to those of control neurons (Fig. 6f–h). Across a range of stimulation intensities, we observed that FCHO1-Δμ-HD neurons exhibited normal endocytic kinetics at low levels of neural activity, but endocytosis became increasingly disrupted under stronger stimulation (Fig. 6k). These results suggest that the μ-HD domain is specifically required for synaptic vesicle endocytosis during prolonged or high-frequency neuronal activity. Collectively, these findings allow us to hypothesize that the μ-HD domain of FCHO1 might plays a critical role in synaptic vesicle endocytosis, particularly during conditions of elevated neuronal activity, whereas it appears dispensable under low-stimulation conditions.

## Discussion

Our study identifies FCHO1 as a critical regulator of synaptic vesicle (SV) endocytosis and reveals stimulation-strength–dependent domain requirements within the protein. Although FCHO1 has been established as a nucleator of clathrin-mediated endocytosis (CME) in non-neuronal systems, its functional contribution at presynaptic terminals has remained incompletely defined. Here, using defined electrical stimulation paradigms and domain-specific rescue strategies, we demonstrate that FCHO1 is indispensable for efficient SV retrieval and that distinct structural domains are differentially required as neuronal activity increases.

Importantly, all stimulation conditions used in this study (25–300 action potentials at 10 Hz) fall within a regime previously established to engage clathrin-mediated endocytosis rather than bulk or ultrafast endocytic pathways [26, 38, 39]. Thus, the differential domain requirements observed here are unlikely to reflect a shift between distinct endocytic mechanisms. Instead, they represent modulation within the CME pathway itself under increasing presynaptic demand.

In this context, increasing the number of action potentials directly increases stimulation strength and, consequently, neural activity. As neural activity rises, the number of synaptic vesicles undergoing exocytosis and therefore requiring retrieval also increases. We interpret our findings within this framework of escalating endocytic load. Under low stimulation (e.g., 25 APs), membrane curvature generation mediated by the F-BAR domain appears sufficient to support vesicle retrieval. However, as stimulation strength increases (100–300 APs), the demand for coordinated recruitment and organization of endocytic machinery becomes greater. Under these conditions, the adaptor-interacting μ-homology domain (μ-HD) becomes essential. Thus, these findings are consistent with modulation within a single endocytic framework rather than engagement of mechanistically distinct pathways, our data support a model in which domain-specific requirements scale with endocytic demand within the CME process.

The domain-deletion experiments are central to this interpretation. While the ΔF-BAR and Δμ-HD constructs localized to presynaptic terminals, deletion-based approaches inherently carry the possibility of structural perturbations or altered protein–protein interactions beyond simple loss-of-function. Although the stimulation-selective nature of the observed phenotypes argues against a generalized dominant-negative effect, we cannot fully exclude subtle structural consequences of domain removal. Therefore, our conclusions regarding domain-specific functions should be interpreted within this experimental limitation.

All functional analyses were performed using vGlut1-pHluorin as a reporter of SV recycling. While cargo-specific differences in recycling kinetics cannot be completely excluded, previous studies using alternative cargo-pHluorin reporters (e.g., synaptophysin-pHluorin, VAMP2-pHluorin, synaptotagmin-pHluorin) have demonstrated broadly similar endocytic kinetics under comparable stimulation paradigms [26, 38, 39]. Thus, although our measurements formally reflect vesicles containing vGlut1, it is unlikely that the observed FCHO1-dependent effects are uniquely restricted to this cargo.

Mechanistically, we propose that μ-HD-dependent regulation reflects increased reliance on adaptor recruitment and molecular scaffolding during high-demand conditions. This interpretation is grounded in prior biochemical and structural studies demonstrating that the μ-HD domain interacts with AP-2 and other adaptor proteins. However, because adaptor recruitment was not directly measured in the present study, this aspect should be considered a model-based inference consistent with existing literature rather than direct mechanistic proof.

From a physiological perspective, differences in stimulation strength may correspond to varying firing patterns in vivo. Sparse or low-frequency activity may impose modest endocytic demand, conditions under which F-BAR-mediated membrane curvature is sufficient. In contrast, sustained burst firing or prolonged high-frequency discharge would markedly increase vesicle turnover, necessitating enhanced adaptor coordination through the μ-HD domain. This scaling model suggests that FCHO1 functions as a demand-sensitive regulator that adjusts CME efficiency according to the magnitude of neural activity.

Beyond neurons, FCHO1 is expressed in multiple tissues, including immune cells. Immune responses likewise vary in magnitude from mild activation to robust inflammatory signaling each imposing distinct membrane trafficking demands. It is therefore conceivable that a similar scaling principle operates in these systems, whereby membrane curvature functions suffice under low-demand conditions, whereas extensive adaptor-mediated scaffolding becomes essential during strong activation. Although speculative, this conceptual extension highlights the broader relevance of stimulation-strength-dependent modularity in multidomain endocytic proteins.

Taken together, our findings establish FCHO1 as an essential component of clathrin-mediated synaptic vesicle endocytosis and support a model in which its structural domains are differentially required as neural activity and thus endocytic demand increases. Rather than functioning as a static nucleator, FCHO1 appears to operate as a demand-sensitive scaffold whose domain-specific contributions scale with the magnitude of synaptic activity.

## Data Availability

No datasets were generated or analysed during the current study.
